# Association myélome multiple – maladie de Kaposi: à propos d’un cas

**Published:** 2010-10-15

**Authors:** Zbiti Najoua, Tarik Houssaini Sqalli, Hakim Hamzaoui, Meriam Meziane, Fouad Zouaidia, Najat Mahassini, Hakima Rhou, Loubna Benamar, Fatima Ezaitouni, Naima Ouzeddoun, Hassam Badreddine, Rabia Bayahia

**Affiliations:** 1 Service de Néphrologie-Dialyse-Transplantation rénale, CHU Ibn Sina, Rabat, Maroc; 2 Service de Dermatologie, CHU Ibn Sina, Rabat, Maroc; 3 Laboratoire d’anatomie pathologique, CHU Ibn Sina, Rabat, Maroc

## Abstract

**Abstract:**

L'association Kaposi–myélome multiple est extrêmement rare. Nous rapportons, le cas d'un patient âgé de 76 ans suivi dans notre formation pour un myélome à immunoglobuline A kappa stade III-B selon Durie et Salmon. Ceci a été associé à des lésions cutanées dont la biopsie cutanée était en faveur d’une maladie de Kaposi. La sérologie de l’herpàs virus humain de type 8 (HHV8) est revenue positive. Une radiothérapie sur les lésions était refusée par le patient. L'évolution était marquée par une altération de l'état général. Le patient ayant refusé la radiothérapie et toute prise en charge thérapeutique est décédée deux mois plus tard. Nous rapportons, à notre connaissance, le 18àme cas mondial de maladie de Kaposi associée à un Kahler chez un patient HHV8 positif. C'est une association exceptionnelle rendant probable le rôle pathogénique de HHV8 dans le développement du myélome.

## Introduction

La maladie de Kaposi est un processus tumoral angiogénique multifocal qui peut être secondaire à une hémopathie. L'association Kaposi-myélome multiple (MM) est extrêmement rare. Nous rapportons le cas d’un patient ayant un MM associé à une maladie de Kaposi, chez qui la sérologie de l’herpès virus humain de type 8 (HHV8) est revenue positive.

## Observation

Mr M.G est âgé de 76 ans, sans aucun antécédent pathologique particulier. Il a présenté une altération de l’état général avec des douleurs osseuses diffuses, ceci a évolué dans un contexte d’amaigrissement important chiffré à 10 kg en un mois. A l’examen clinique, on a découvert des lésions nodulaires violacées au niveau des deux jambes et des deux pavillons de l’oreille. Au bilan radiologique, on a trouvé des lésions osseuses lytiques diffuses avec des lacunes au niveau du crâne.

Le bilan biologique a objectivé un syndrome inflammatoire (la vitesse de sédimentation globulaire était à 112 mm à la première heure, la protéine C réactive à 7 mg/L, une anémie à 9 g/dL normochrome normocytaire); une neutropénie à 1000/mm3, une lymphopénie à 400/ mm3, une hypercalcémie à 135 mg/l, une insuffisance rénale aigue avec une créatinine sérique à 24 mg/L. L’électrophorèse des protides plasmatiques montrait un pic d’aspect monoclonal au niveau des gammaglobulines à 22 g/L. L’immunoélectrophorèse des protides plasmatiques a montré une immunoglobuline (Ig) A kappa monoclonale. La protéinurie était à 12 g/24 h faite de chaînes légères libres. Le myélogramme mettait en évidence une plasmocytose à 60 %. Il s’agissait donc d’un MM à IgA kappa stade III-B de Durie et Salmon avec une atteinte rénale de type tubulopathie myélomateuse.

Un traitement a été instauré, en urgence, à base de réhydratation et d’alcalinisation associé au biphosphonates. Vue l’atteinte hématologique, un traitement à base de déxaméthasone a été démarré à raison de 40mg/j pendant 4 jours.

**Figure 1:**
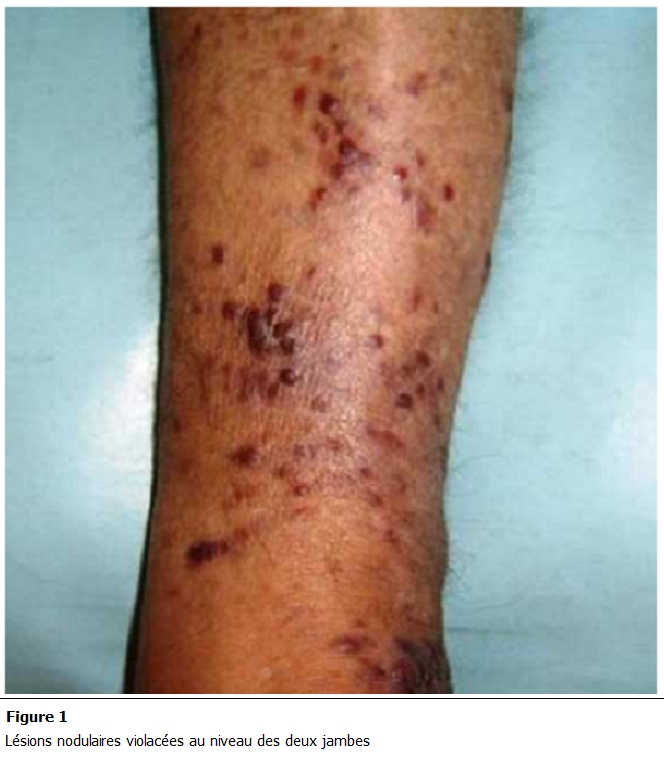
Lésions nodulaires violacées au niveau des deux jambes

**Figure 2:**
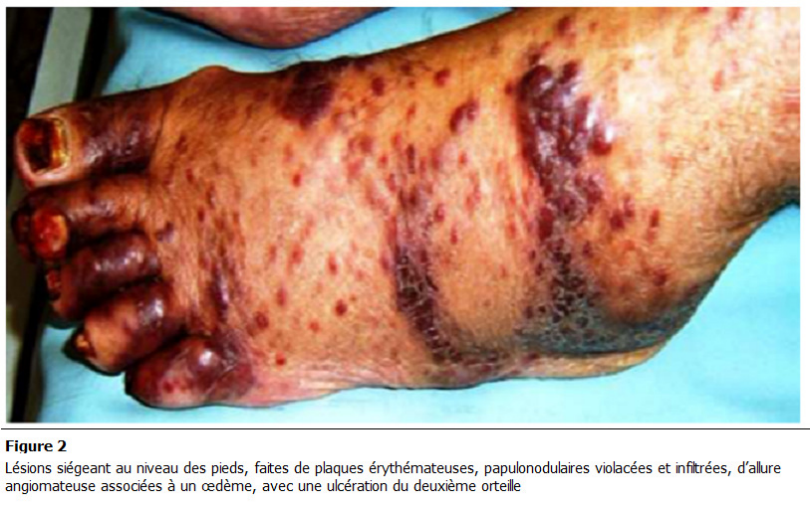
Lésions siégeant au niveau des pieds, faites de plaques érythémateuses, papulonodulaires violacées et infiltrées, d’allure angiomateuse associées à un œdème, avec une ulcération du deuxième orteil.

**Figure 3:**
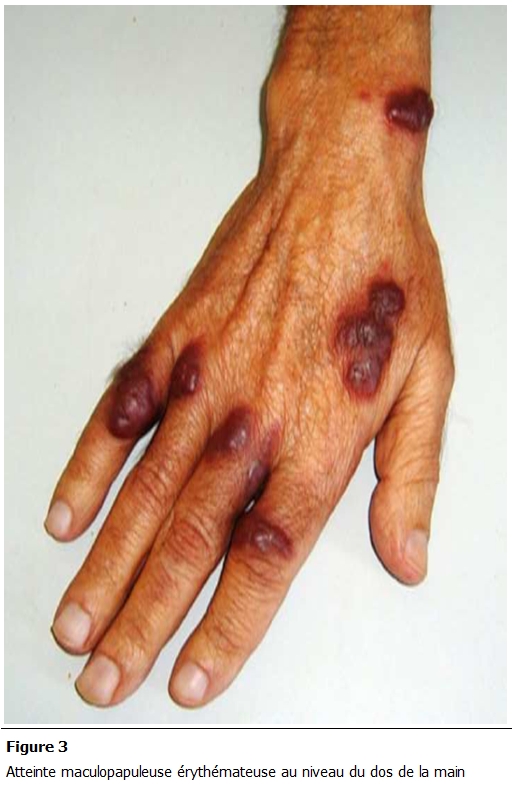
Atteinte maculopapuleuse érythémateuse au niveau du dos de la main

L’évolution a été marquée par une légère amélioration biologique. Par ailleurs, on notait une extension des lésions cutanées avec des plaques érythémateuses papulonodulaires violacées et infiltrées, d’allure angiomateuse. Ces lésions prédominaient au niveau des extrémités où elles étaient associées à un œdème (Figure 1,2,3).

**Figure 4:**
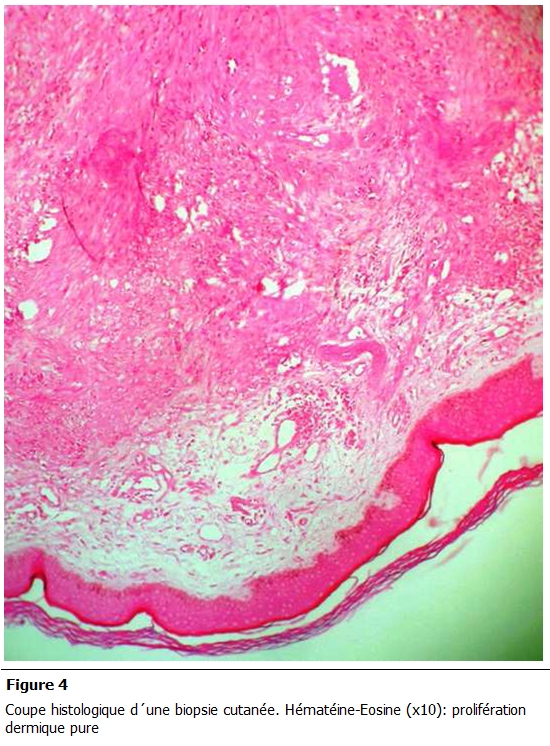
Coupe histologique d’une biopsie cutanée. Hématéine-Eosine (x10): prolifération dermique pure

**Figure 5:**
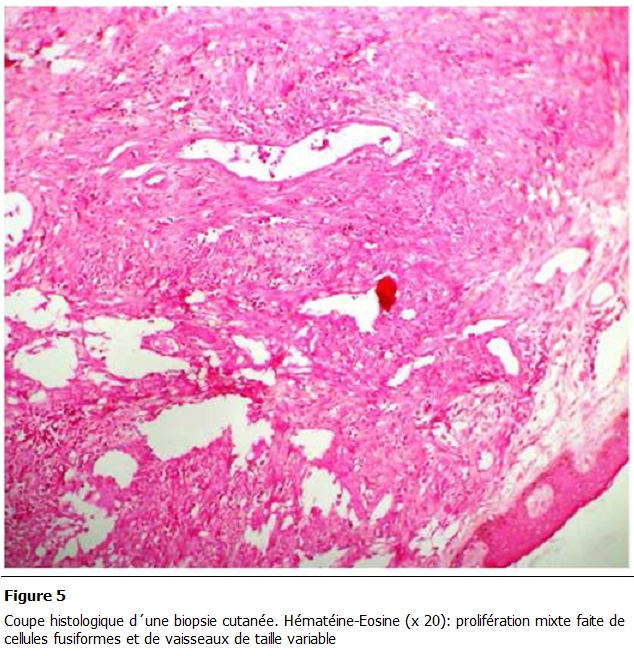
Coupe histologique d’une biopsie cutanée. Hématéine-Eosine (x 20): prolifération mixte faite de cellules fusiformes et de vaisseaux de taille variable

Une biopsie cutanée a été réalisée. Des lésions érythémateuses sont apparues au niveau de la muqueuse buccale gingivale, des organes génitaux externes (gland). Il n’y avait pas d’adénopathies périphériques, et le bilan d’extension a écarté une atteinte viscérale. L’examen histologique a porté sur un revêtement cutané dont le derme était siège d'une prolifération tumorale faite de structures vasculaires, de tailles variables, intercalées par des faisceaux de cellules fusiformes aux noyaux modérément atypiques et avec quelques figures de mitoses. Le diagnostic retenu était une maladie de Kaposi (Figure 4,5).

La sérologie de HHV8 est revenue positive, quant aux sérologies des virus d’Epstein-Barr, cytomégalovirus, hépatite B et C, et VIH étaient négatives.

Une radiothérapie sur les lésions était refusée par le patient. Le patient est décédé deux mois plus tard dans un contexte d’altération de l’état général.

## Discussion

Le sarcome de kaposi est une entité anatomoclinique très particulière. C’est une maladie mésenchymateuse systémique, multifocale dont les localisations cutanées dominent le tableau. C’est en 1872 que le dermatologue autrichien “Moricz Cohen Kaposi” décrivait pour la première fois la maladie qui porte son nom sous le terme de “Sarcoma Multiplex Idiopathicum”. Sa physiopathologie, intriquée à celle d’un nouvel herpèsvirus (HHV8) découvert en 1994 [1], passionne de nombreux chercheurs. Cette maladie, autrefois rare, s’est répandue dans les années 1980, en même temps que l’épidémie du syndrome de l’immunodéficience aquise (sida) ([1,2].

L’association maladie de Kaposi-affection hématologique est connue et concerne surtout les lymphomes, hodgkiniens ou non. La maladie de Kaposi précède l’hémopathie dans 15 % des cas, elle apparaît simultanément dans 36 % des cas et après dans 46 % des cas [3,4]. Dans notre cas, la découverte des lésions cutanées concomitait avec celle de l’atteinte hématologique.

La maladie de Kaposi fait partie des tumeurs induites par les virus. Elle est, très probablement, due à une infection chronique par le HHV8. En effet la séroconversion précède les lésions de Kaposi de 33 mois en moyenne [1].

En 1997, l’équipe de Retting. [5] a détecté de l’ADN viral d’HHV8 dans les cellules dendritiques et les cellules mononuclées de la moelle osseuse de sujets atteints d’un MM. Notre malade avait une maladie de Kaposi associée à un MM, et la recherche de HHV8 est revenue positive. Le HHV8 code pour un homologue viral de l’interleukine-6 (IL-6) capable d’induire la survie et la prolifération plasmocytaire [6-9].

En 1998 l’équipe de J. Berenson a pu démontrer que, chez les malades atteints de gammapathies monoclonales, le virus HHV8 infecte un type particulier de cellules dendritiques stromales médullaires adhérentes à l’os et productrices d’IL-6v. Les cellules infectées sont présentes en très faible quantité dans les aspirations médullaires où elles sont indécelables mais s’amplifient fortement in vitro. Cette infection jouerait un rôle majeur non seulement dans l’émergence du MM mais aussi dans son évolution clinique et serait donc à prendre en compte lors de la prise en charge des patients [10,11].

L'atteinte viscérale fait toute la gravité de la maladie de Kaposi, en particulier l'atteinte pulmonaire.

Le traitement local est indiqué dans le cas d'atteintes cutanéomuqueuses stables et limitées. Un traitement systémique est indiqué en cas de lésions étendues, d’évolutivité rapide, d’œdèmes douloureux segmentaires ou d’atteinte viscérale.

L’expérience de l’interféron dans la maladie de Kaposi classique est plus limitée qu’au cours du sida, mais elle est extrêmement encourageante.

Le pronostic est corrélé à l’état immunitaire du patient, et non au nombre de lésions [2]. Notre patient avait un MM actif avec une masse tumorale élevée, le pronostic est devenu plus mauvais devant l’extension de la maladie de kaposi et son refus de toute prise en charge thérapeutique.

## Conclusion

Nous avons rapporté, à notre connaissance, le 18^ème^ cas de maladie de Kaposi associée à un MM. C'est une association exceptionnelle rendant probable le rôle éventuel de HHV8 dans le développement du myélome, ce concept deviendrait un élément essentiel pour progresser dans la physiopathologie et le traitement de cette maladie.

## Conflit d’intérêts

Les auteurs ne déclarent aucun conflit d’intérêts

## Contribution des auteurs

Tous les auteurs ont également participé a la prise en charge du patient et a la rédaction du manuscrit. La version finale a été revue et approuvé par tous les auteurs.
